# Azadiradione ameliorates polyglutamine expansion disease in *Drosophila* by potentiating DNA binding activity of heat shock factor 1

**DOI:** 10.18632/oncotarget.12930

**Published:** 2016-10-26

**Authors:** Vinod K. Nelson, Asif Ali, Naibedya Dutta, Suvranil Ghosh, Manas Jana, Arnab Ganguli, Andrei Komarov, Soumyadip Paul, Vibha Dwivedi, Subhrangsu Chatterjee, Nihar R. Jana, Subhash C. Lakhotia, Gopal Chakrabarti, Anup K. Misra, Subhash C. Mandal, Mahadeb Pal

**Affiliations:** ^1^ Division of Molecular Medicine, Bose Institute, Kolkata, West Bengal, India; ^2^ Dr. B. C. Guha Center for Genetic Engineering and Biotechnology, University of Calcutta, Kolkata, West Bengal, India; ^3^ Cellecta Inc, Mountain View, California, United States of America; ^4^ Department of Zoology, Cytogenetics Laboratory, Banaras Hindu University, Varanasi, Uttar Pradesh, India; ^5^ Department of Biophysics, Bose Institute, Kolkata, West Bengal, India; ^6^ Cellular and Molecular Neuroscience Laboratory, National Brain Research Institute, Manesar, Gurgaon, Haryana, India; ^7^ Department of Pharmaceutical Technology, Pharmacognosy and Phytotherapy Laboratory, Jadavpur University, Jadavpur, West Bengal, India

**Keywords:** heat shock factor 1, HSF1, neurodegenerative diseases, small molecule, azadiradione, Gerotarget

## Abstract

Aggregation of proteins with the expansion of polyglutamine tracts in the brain underlies progressive genetic neurodegenerative diseases (NDs) like Huntington's disease and spinocerebellar ataxias (SCA). An insensitive cellular proteotoxic stress response to non-native protein oligomers is common in such conditions. Indeed, upregulation of heat shock factor 1 (HSF1) function and its target protein chaperone expression has shown promising results in animal models of NDs. Using an HSF1 sensitive cell based reporter screening, we have isolated azadiradione (AZD) from the methanolic extract of seeds of *Azadirachta indica,* a plant known for its multifarious medicinal properties. We show that AZD ameliorates toxicity due to protein aggregation in cell and fly models of polyglutamine expansion diseases to a great extent. All these effects are correlated with activation of HSF1 function and expression of its target protein chaperone genes. Notably, HSF1 activation by AZD is independent of cellular HSP90 or proteasome function. Furthermore, we show that AZD directly interacts with purified human HSF1 with high specificity, and facilitates binding of HSF1 to its recognition sequence with higher affinity. These unique findings qualify AZD as an ideal lead molecule for consideration for drug development against NDs that affect millions worldwide.

## INTRODUCTION

Neurodegenerative diseases (NDs) like polyglutamine (polyQ) based diseases which include spinal and bulbar muscular atrophy (SBMA), dentato-rubral-pallidoluysian atrophy (DRPLA), Machado-Joseph disease MJD/SCA3), several spinocerebellar ataxias (SCA), and Huntington's, Parkinson's- and Alzheimer's diseases affect millions of people worldwide [1, 2]. People affected with these diseases survive in a debilitated condition, which imposes a heavy financial and psychological burden on the society. Till date, there is no definitive cure as the current treatment options offer only disease-specific management strategies such as neuroleptic and antipsychotic drugs for temporary relief of disease symptoms [3, 4].

Accumulation of non-native protein aggregates, defective cellular heat shock response (HSR) and compromised protein quality control pathways are common hallmarks of various NDs [5–8]. In normal cells heat shock factor 1 (HSF1), the central regulator of HSR, is sequestered in the cytoplasm in a repressive complex assembled with HSP90, p23, immunophilin, HSP70, and HSP40 [9–12]. Exposure to elevated temperature or a proteotoxic stress results in the disassembly of this repressive complex so that the released monomeric HSF1 molecules assemble into DNA binding competent homotrimer form. HSF1 as a homotrimer binds to the recognition element HSE (repetitive 5′-nGAAn-3′) on its target chaperone genes to activate their expression which helps in refolding of mis- or unfolded proteins or removal of the non-native protein aggregates [13]. Involvement of ribonucleoprotein complexes in the trimerization and activation of HSF1 has been demonstrated [14, 15]. Chemical inhibition of HSP90 and proteasome also results in HSF1 activation [12, 16].

Forced upregulation of HSF1 and or its target genes such as HSP70 was shown to reduce protein aggregate accumulation, associated toxicity, and diseases symptoms in C. elegans and mouse models of Huntington's disease [5, 6, 17]. As expected HSF1 downregulation was also associated with enhanced polyglutamine-induced toxicity [18]. Several small molecule activators of HSF1 obtained through screening of small molecule libraries and natural products, have been reported and are in various developmental stages of drug development [3, 19]. Nevertheless, given the severity of disease burden on the patients and the society, compounds with novel and unique functional mechanisms are desired in the repertoire to explore better treatment options. A small molecule with a unique mode of action is also desired to obtain deeper insights into the molecular mechanisms of function of HSF1.

We report here azadiradione (AZD) as an inducer of HSF1 activity. We obtained AZD through screening of methanolic extract of *Azadirachta indica* seeds by cell-based reporter activity assay coupled purification approach. *Azadirachta indica*, locally known as Neem, has been in use in the traditional medicine for treatment of many diseases because of its anti-inflammatory, anti-anxiety activities, and for enhancement of cognitive ability [20, 21]. We show here that treatment with AZD efficiently ameliorates polyglutamine (polyQ) protein induced toxicity in the cellular and fruit fly models. The ameliorating effects were correlated with AZD induced upregulation of heat shock protein chaperone (HSP) expression mediated by direct interaction with HSF1 protein.

## RESULTS

### Identification and purification of azadiradione from neem seeds as an activator of HSF1 by cell based reporter assays

**Figure 1:**
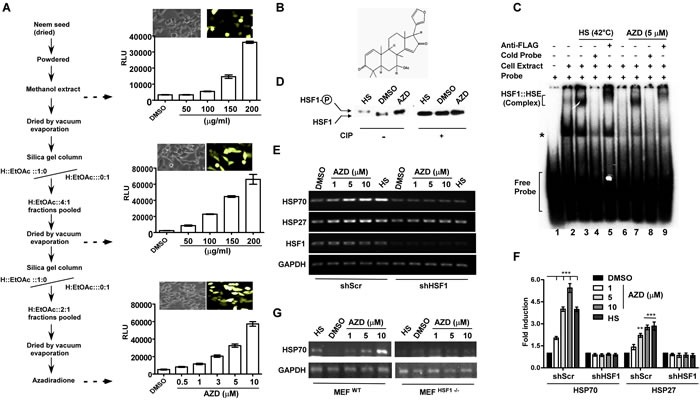
Azadiradione (AZD) activates HSF1 and its target heat shock chaperone genes **A.** Flowchart of stepwise purification of AZD accompanied by an increase in specific activity measured by GFP expression (next to phase contrast images of cells) and luciferase assays as indicated. H, n-hexane; values are mean±SEM, *n* ≥ 3. **B.** Chemical structure of AZD. **C.** AZD induces DNA-binding ability of HSF1 in cells determined by EMSA using γ-^32^pATP-labeled HSE (~1 ng/reaction) as described in the materials and methods. Whole cell extract (WCE) (50 μg) prepared from HEK293 cells expressing FLAG-HSF1 pre-treated with AZD or DMSO or HS (at 42°C/1 h) were used as indicated. The reactions were resolved on a 4% acrylamide and bis-acrylamide (29:1) gel and autoradiographed. **D.** AZD treatment induces HSF1 hyperphosphorylation like HS in WCE used in (C) which was erased upon phosphatase treatment determined by immunoblot with α-FLAG antibody. **E.** AZD treatment induces HSP70 and HSP27 transcription by HS in HEK293 cells that is sensitive to shRNA-mediated HSF1 downregulation (shHSF1) as determined by RT-PCR assay. Representative agarose gels of PCR products obtained using equivalent amounts of cDNAs as templates prepared from transcripts isolated from cells treated as indicated. **F.** Densitometric quantitation of HSP70 and HSP27 bands in agarose gels as shown in panel E from three independent experiments. **p < 0.01;***p < 0.001. **G.** AZD activates HSP70 only in MEF cells carrying wild type HSF1 MEF^WT^ gene but not in MEF cells that have been deleted both copies of HSF1 gene (MEF^HSF1−/−^). Expression of HSP70 mRNA was measured by semiquantitative RT-PCR assay. The PCR products were resolved in agarose gels visualized by ethidium bromide staining.

Screening for activator of HSF1 was carried out using cell-based reporter system harboring renilla luciferase (Rluc) and GFP independently under the control of six tandem copies of HSE (6xHSE). The reporter constructs were stably integrated into the genome of HCT116 cells by lentiviral transduction [22, 23]. Incubation of the reporter cells with the methanolic extract of neem seeds resulted in the activation of both GFP and Rluc reporters in a dose-dependent manner (Figure 1A). To understand the nature of the compound, the activity was purified by several rounds of silica gel chromatography using solvents of various polarities to collect the most active fraction. The purification was verified by an increase in the specific activity by Rluc assay at each purification step (Figure 1A). Finally, an active molecule was purified, and the obtained compound was found to be more than 95% pure by thin layer chromatography (TLC) and high-performance liquid chromatography (HPLC) techniques. Electrospray ionization (ESI) mass spectrometer analysis indicated the mass of the compound to be 450.7953 Dalton (Figure S1A). The molecular structure of the compound as azadiradione (AZD) was confirmed by analyzing the data obtained from ESI mass- and ^1^H and ^13^C NMR spectroscopy as well as by analytical HPLC analyses (Figure 1B, S1A-C).

### Azadiradione induces HSF1 activity in cells

Hyperphosphorylation, ability to bind the HSE, and activation of transcription of chaperone genes are signatures of HSF1 activation process [24]. To test the activity of HSF1, whole cell extract (WCE) of HEK293 cells pre-treated with AZD or DMSO or heat shock (HS, as positive control), were prepared. The HEK293 cells used here carried a stably expressed FLAG-tagged HSF1 protein to monitor HSF1 through anti-FLAG antibody (Figure S3G). We did not see any change in the HSF1 sensitivity to heat shock or AZD due to its overexpression (data not shown). As revealed by electrophoretic mobility shift assay (EMSA), WCE prepared from AZD or HS treated cells showed enhancement of HSE binding to similar level (Figure 1C, compare lanes 1-3 & 7). Formation of a supershifted complex by addition of anti-FLAG antibody indicated the presence of FLAG-HSF1 in the complex (Figure 1C, compare lanes 3 & 5, and 7 & 9). (The band moving faster than the HSE-HSF1 complex as shown by an asterisk may be indicative of a complex formed on the HSE by Ku70/80 in the absence of heat shock as reported earlier [25]). Activation of HSF1 by AZD in the cell was indicated by hyperphosphorylation of HSF1 compared to vehicle treated sample as estimated by relative retardation in mobility during electrophoresis in a denaturing polyacrylamide gel detected by immunoblotting. A phosphatase (CIP) treatment led to collapse of slower moving HSF1 species to a relatively faster moving species like that in vehicle treated samples (Figure 1D). A transcriptional activation of HSF1 by AZD treatment was indicated by dose-dependent increase in the expression of its targets HSP70A1A (denoted as HSP70 in all subsequent experiments) and HSP27. Like the conventional heat shock [19, 26–28], AZD treatment provided cytoprotection against lethal heat shock stress (45°C for 1 h) in a dose-dependent manner (Figure S2A). Results of two indepentdent experiments support that AZD mediates its function through HSF1: a) shRNA-mediated downregulation of HSF1 reduced expression of HSPs by AZD treatment compared to scrambled shRNA treated cells (Figure 1E-1F) and b) AZD failed to induce HSP70 in mouse embryonic fibroblast (MEF) cells where HSF1 was completely deleted (MEF^HSF1−/−^) while MEF cells carrying normal HSF1 gene (MEF^WT^) induced HSP70 as usual (Figure 1G).

### Azadiradione reduces protein aggregate formation in cell model of polyglutamine disorder

Classical heat shock response (HSR) upregulates expression of HSPs that are capable of refolding of unfolded or misfolded to their native state in the cell [29]. HSR can also activate ubiquitin-dependent proteasome pathway that helps clear accumulation of protein aggregate in cells [5, 16].

**Figure 2:**
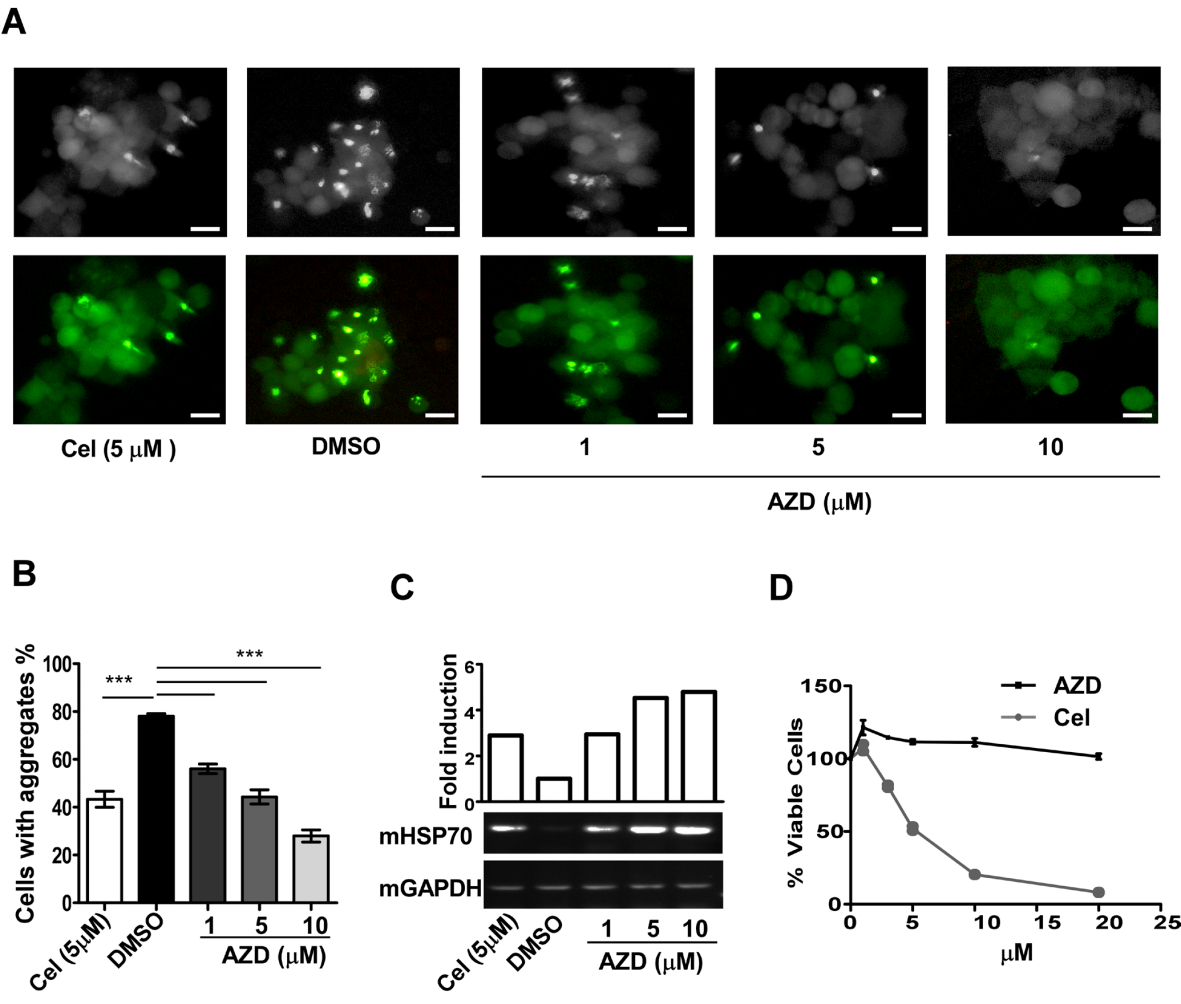
AZD reduces protein aggregation and associated toxicity in the cell **A.** Effect on ataxin130Q-GFP aggregation by AZD, or celastrol, (Cel) or DMSO as indicated in Neuro-2a cells. Scale bar 10 μm. **B.** The aggregates were counted and plotted after 48 h treatment, 450 cells were counted for each treatment, ***p < 0.001. **C.** Ethidium bromide stained agarose gel showing relative levels of HSP70 (mHSP70) transcripts isolated from cells subjected to identical treatments in duplicate as represented in panel (A) estimated by semiquantitative RT-PCR assays. **D.** Comparison of sensitivity of Neuro-2a cells to AZD or celastrol after 24 h treatment estimated by MTT assay. Data are mean±SEM, *n* ≥ 3

AZD was tested for its ability to reduce levels of aggregation of an aggregation-prone ataxin130Q-GFP fusion protein transiently expressed in mouse neuroblastoma (Neuro-2a) cells compared to vehicle treated samples. Cells grown to ~50% confluency were treated with DMSO (vehicle control), three different concentrations of AZD or celastrol (positive control) for 48 h. To correlate the results with chaperone function, cells are treated identically in duplicate for estimating the HSP70 transcript levels under the different treatment conditions. Results showed that with increasing concentration of AZD there was a gradual decrease of ataxin130Q-GFP aggregate levels in cells as measured by GFP fluorescence. Interestingly, AZD action in reducing the ataxin130Q-GFP aggregate levels was better than that of celastrol (positive control). In agreement with the reduction in aggregation, HSP70 transcript levels were also increased in AZD treated samples in a dose-dependent manner, indicating a correlation between the AZD mediated protein aggregation resolving activity and elevation of HSP70 expression (Figure. 2A-2C) [30]. To compare the relative toxicity of AZD with celastrol, Neuro-2a cells were treated with increasing doses of these two compounds for 24 h followed by measuring the fraction of surviving cells using the MTT assay. The AZD was found to be relatively less toxic than celastrol (Figure 2D). Sensitivity slightly increased in 72 h assay condition; about 20% cell died in 10 μM AZD compared to 80% by celastrol (Figure S2B). Celastrol has been shown to work through inhibition of HSP90 as well as proteasome function [26, 31].

### Effect of azadiradione on fruit fly model of poly-Q disease

**Figure 3:**
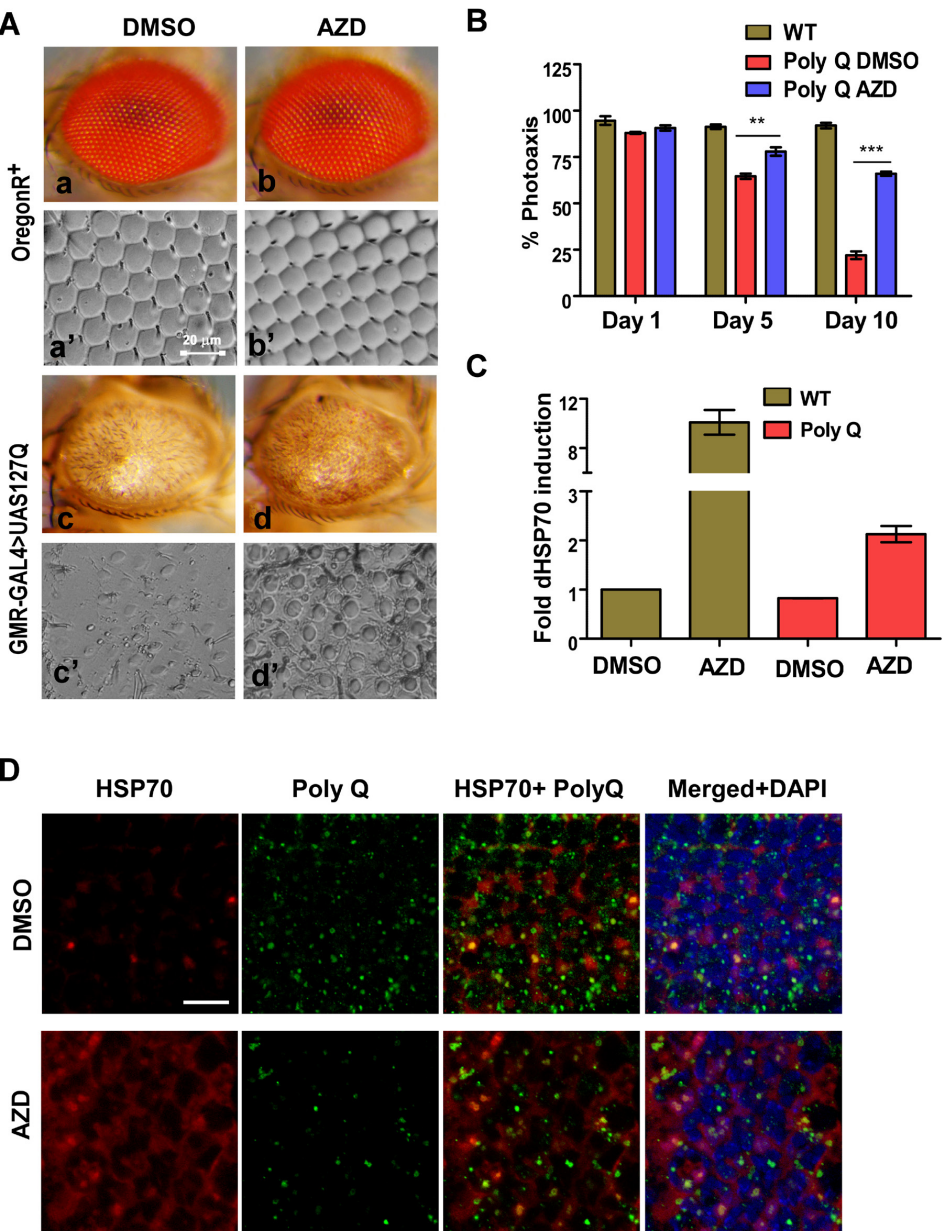
AZD feeding ameliorates 127Q (polyQ) induced eye defect and vision in the fruit fly **A.** Dietary supplementation of AZD improved eye morphology and structure of ommatidial arrays (a-c' vs. b-d') damaged by polyQ expression. **B.** AZD supplementation significantly improved the vision defect caused by polyQ expression in the fly eyes as determined by phototaxis assay. Shown are the proportion (% on Y-axis; N = 75 flies in each case) of wild type and polyQ expressing flies (as indicated on the top) moving to the illuminated chamber on different days (X-axis; ** and *** indicate *P* < 0.01 and < 0.001), respectively, for comparison between DMSO and AZD treated polyQ flies. **C.** Dietary supplementation of AZD induces levels of HSP70 transcripts (Y-axis) in the head of the flies not expressing or expressing polyQ (X-axis). Data are mean±SEM, *n* ≥ 3. **D.** Compared to DMSO (upper row) dietary supplementation of AZD induces expression of HSP70 and reduces the level of polyQ in retinal cells as detected by immunostaining as indicated. Individual or merged staining in the confocal projection images are indicated on top of each column. Scale bar 20 micrometer.

A polypeptide carrying expanded polyglutamine repeats (127Q residues/127Q) under UAS promoter was over-expressed predominantly in the larval eye imaginal discs of fruit flies using the eye-specific *GMR-GAL4* driver [32]. An overexpression of the protein resulted in the retinal degeneration and defective eye development in the adult fly (Figure 3A). *GMR-GAL4 > UAS-127Q* and wild type OregonR+ larvae were grown on food supplemented with AZD or DMSO (vehicle control) from 1^st^ instar larval stage to adult stage to examine if AZD has any effect on retinal development in the 127Q expressing flies. We examined adult fly eye morphology as well as the organization of the ommatidial arrays through the nail polish imprints. 127Q overexpression in the vehicle control (DMSO-fed) flies resulted in severe degeneration of retina including near complete disorganization of ommatidial arrays [33]. Interestingly, AZD supplementation suppressed the damage to these structures significantly so that some indications of ommatidial arrays were distinctly noticeable (Figure 3A: c vs d and c' vs d'). The wild type Oregon R+ larvae continuously fed on either AZD or DMSO alone till adult stage developed normal eye morphology with the characteristic ommatidial array architecture. These results indicate that AZD is not toxic to the flies (Figure 3A, a vs. b, and a' vs. b').

We next examined the functionality of eyes through phototaxis-assay which provides a good estimate of vision of flies as they are provided a choice between illuminated and dark chambers. The *GMR-GAL4 > UAS-127Q* expressing 1 day, 5 days or 10 days old adult flies, fed as larvae on AZD or DMSO supplemented food, were subjected to phototaxis assay. As shown in Figure 3B, DMSO-treated 127Q expressing flies were almost blind by day 10 while those reared on AZD supplemented food retained fairly good vision as a majority of them (~75% of age-matched wild type flies) perceived light and moved to the illuminated chamber during the assay period [33]. To examine if the recovery in the retinal damage and vision following AZD treatment was related to elevated HSP70 expression, qPCR was carried out using the cDNA prepared from the total RNA isolated from fly heads. Expression of HSP70 transcripts was significantly elevated in AZD treated samples compared to DMSO, in wild type as well as in 127Q expressing samples. A good correlation between HSP70 and the suppression of polyQ damage (Figure 3C-3D) suggests that AZD reduced polyQ toxicity through induction of chaperones. Eye discs from AZD or DMSO fed 127Q expressing larvae were immunostained for HSP70 and polyQ inclusion bodies. As shown, AZD treatment resulted in a robust induction of HSP70 protein and suppression of 127Q aggregates when compared with those from DMSO-treated larvae (Figure 3D). HSP70 overexpression and associated effect appeared to be due to activation of HSF by AZD treatment rather than due to transcriptional induction of HSF gene [6] since qPCR analysis of cDNA samples used in panel C did not show any increase in levels of of HSF transcripts in AZD treated samples (not shown). Thus, we believe that AZD treatment restrained polyQ induced damage in fly eye through activation of HSF and the consequent elevated expression of HSP70 protein.

### Azadiradione does not interfere with proteasome or HSP90 activity

Inhibition of ubiquitin-proteasome and HSP90 activities also results in HSF1 activation [13]. Both of these activities that are involved in either refolding, or degradation and clearance of unfolded proteins are essential in a healthy cell [16]. Small molecule activators of HSF1 identified so far were shown to function either by inhibition of HSP90, proteasome or TRiC/CCT [3].

**Figure 4:**
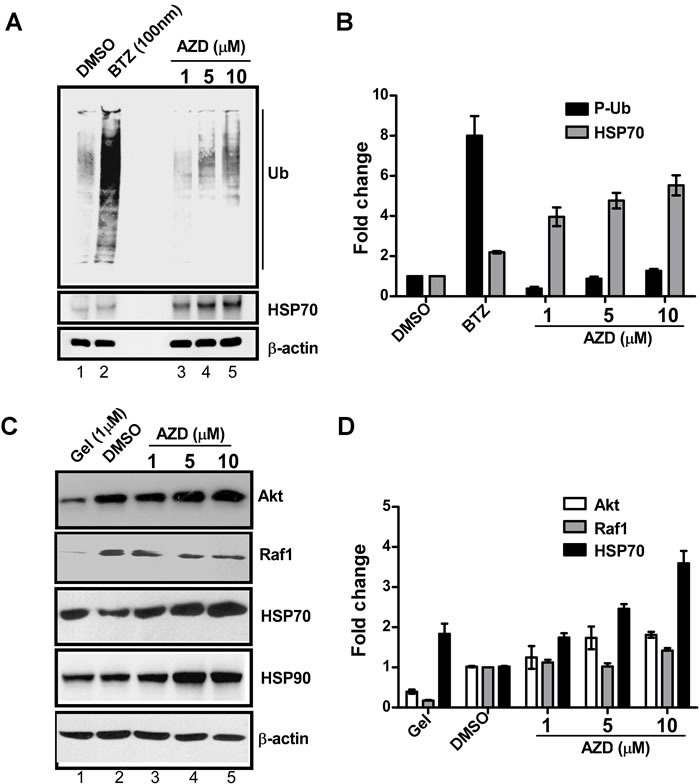
AZD induces HSP70 protein expression without interfering with the functions of HSP90 or proteasome WCE (20 μg) of HEK293 cells pretreated for 16 h with bortezomib (BTZ) or geldanamycin (Gel) or various concentrations of AZD, or DMSO were subjected to immunoblot **A.** using the antibodies against anti-ubiquitin (P-Ub). The levels of HSP70 protein in the same samples were also determined as indicated using anti-HSP70 antibody. **B.** The bands in the blot were estimated and plotted as bar graph considering ß-actin levels as an internal loading control. **C.** Immunoblot with antibodies against two HSP90 client proteins Akt or Raf1 and HSP70 and HSP90 itself to compare their relative expression levels in those samples. **D.** Band intensities in the blot were estimated by densitometric scanning and plotted to compare their expression levels. ß-actin levels were used as internal loading control.

To test effect of AZD on proteasome function, HEK293 cells were treated with three concentrations of AZD, or DMSO or a known proteasome inhibitor bortezomib (as a positive control) for 16 h and levels of poly-ubiquitinated proteins (P-Ub) were estimated by immunoblotting. Relative inductions of HSP70 protein levels were also measured in these samples to correlate proteasome inhibition with the HSF1 activation (Figure 4A). Comparison of relative proteasome activity versus HSP70 expression between AZD and bortezomib treated samples revealed that proteasome activity after AZD treatment increased slightly though gradually while HSP70 protein levels were enhanced in dose-dependent manner. Bortezomib treatment did not show a significant induction of HSP70 while showed a huge increase in P-Ub signals (Figures 4A-4B). Low sensitivity of HSP70 transcription in response to bortezomib was also observed by others in HEK293 [23, 34]. Therefore, it appears that the elevated HSP70 expression following AZD treatment is independent of proteasome function.

To test the effect on HSP90 activity HEK293 cells were treated with three concentrations of AZD or DMSO, or geldanamycin which is a known HSP90 inhibitor (as the positive control) (Figure 4C). Relative stabilities of HSP90 client proteins, such as Akt and Raf1 in whole cell extracts were estimated by western blotting. As the data in Figure 4C-4D show, levels of both the Akt and Raf1 client proteins were significantly reduced in geldanamycin treated WCEs. However, the WCE from AZD treated cells showed little change in the levels of these two proteins, even with increasing concentrations of AZD. The levels of HSP70 and HSP90 proteins in geldanamycin and AZD treated samples increased in dose dependent manner (Figure 4C-4D). These results indicate that the elevated level of HSP70 in AZD cells do not involve HSP90 activity.

### Azadiradione facilitates DNA binding by direct physical interaction with HSF1

Conversion of monomeric HSF1 present in the cytoplasm into a DNA-binding competent homotrimeric state is believed to be an important step in its activation pathway [24]. Purified HSF1 *in vitro* has also been shown to intrinsically form homotrimer, which is competent to bind to its recognition (HSE) sequence in response to heat shock or other stressful conditions [35–37]. The importance of two cysteine residues in the DNA-binding domain of HSF1 was shown by mutational analysis [36].

**Figure 5:**
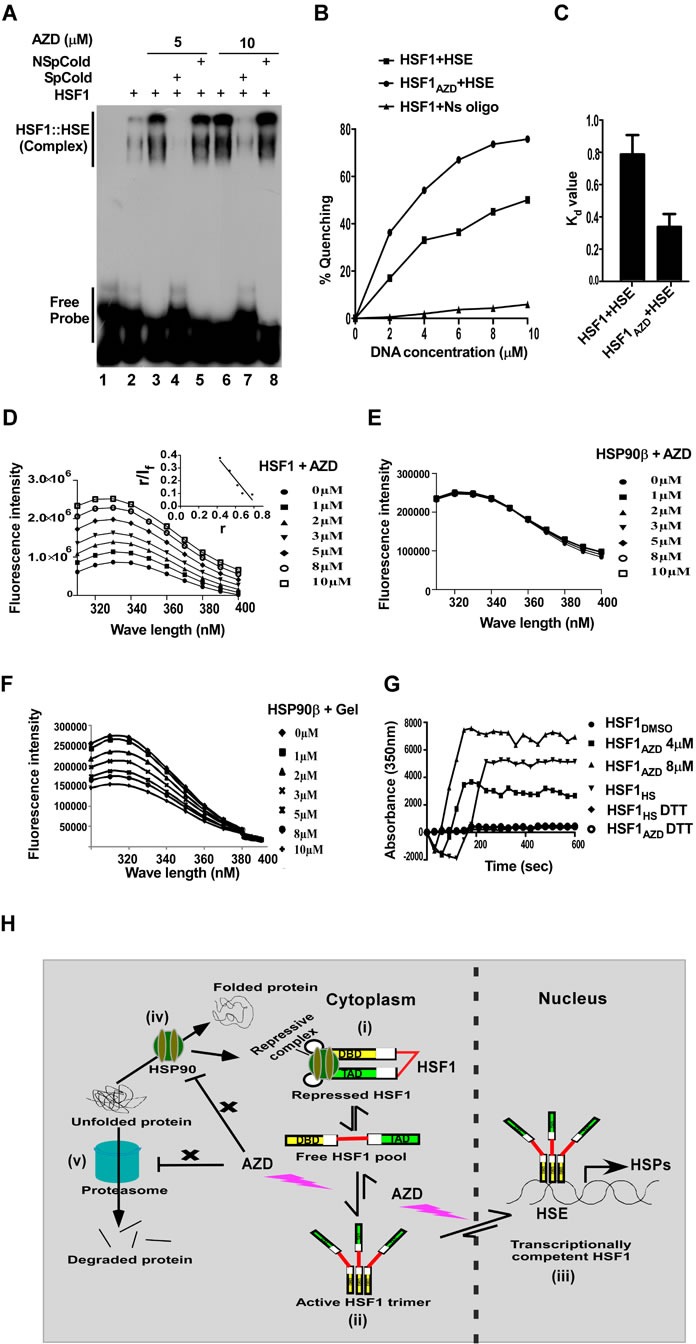
AZD directly interacts with the purified HSF1 protein tested by EMSA as well as fluorometric assay **A.** Autoradiogram of an EMSA showing concentration-dependent enhancement of radiolabeled HSE binding of HSF1 (0.5 μM) by AZD. The binding is competed out by excess unlabeled HSE (SpCold) but not by an unrelated double-stranded (ds) DNA oligonucleotide (NSpCold) as described in the method. **B.** Effect of AZD (5 μM) on the binding of increasing concentration HSE by HSF1 (2 μM) measured by fluorometric assay as indicated. **C.** Bar diagram representing the Kd values of HSF1 binding to HSE without or with AZD. **D.** Concentration dependent effect of AZD as indicated on HSF1 (2 μM) fluorescence (inset: Scatchard plot of AZD binding to HSF1) and **E.** on HSP90 (2 μM) fluorescence. **F.** Effect of geldanamycin (Gel) on the indicated concentration on HSP90 fluorescence. **G.** Concentration dependent effect of AZD (as indicated) or heat shock (HS) on HSF1 (2 μM) multimerization in the cell-free system determined by monitoring the kinetics of polymerization by using light scattering at 350 nm. The presence of DTT in the reaction abolished the polymerization induced by AZD or HS. **H.** Cartoon proposing AZD targeting the free pool of monomeric HSF1, an intermediate between repressed (i) and active HSF1 (homotrimeric state) (ii) in the cell that translocates to the nucleus to execute its transcription function (iii). HSF1 is kept in inactive monomeric state by a repression complex composed of proteins including HSP90, HSP70, p23 and immunophilin (i); AZD does not disturb (indicated by a cross symbol) HSP90 chaperone function of refolding of unfolded/misfolded client proteins (iv), or proteasome that degrades irreversibly unfolded protein (v).

As noted above, AZD did not interfere with the function of proteasome or HSP90 in cells. Therefore, we examined if AZD influences DNA binding efficacy of HSF1 (Figure 5). Human HSF1 purified from overexpressing bacterial strain (Figure S3E) was incubated with radio-labeled HSE (ds DNA oligonucleotide) either in the presence of DMSO or increasing concentrations of AZD. Most interestingly, the gel mobility shift assay (EMSA) clearly revealed increased binding of HSF1 in the presence of AZD in a concentration dependent manner (Figure 5A, lanes 1-3 and 6). AZD increased the HSF1 binding affinity to HSE by more than two-fold (also see Figure S3F). The specificity of the interaction was confirmed by the elimination of the binding by addition of an excess unlabeled double stranded HSE (Figure 5A, lanes 3-4, and 6-7) but not by an unrelated dsDNA oligonucleotide of similar molecular weight (Figure 5A lanes 3-5 and 6-8). These results strongly suggest that AZD specifically facilitates DNA binding of HSF1 *in vitro*.

This property of AZD was re-evaluated by fluorometric assay. Binding of HSE quenched fluorescence intensity of HSF1 (tryptophan fluorescence) with an increase in the concentration of DNA (0-10 μM). The dissociation constant (Kd) of the HSE and HSF1 interaction was estimated by measuring the quenching of HSF1 fluorescence. The titration curve of a constant amount of HSF1 (2 μM) and various concentrations of HSE (0-10μM) was analyzed by the double reciprocal plot as described in Experimental Procedures. This analysis yielded a linear plot with a dissociation constant of 0.8 μM. The binding was very specific as little change in tryptophan fluorescence of HSF1 was observed when it was incubated with an unrelated dsDNA oligonucleotide (HSF1+Ns oligo) (Figure 5B-5C, S3A-B). We next examined the effect of AZD on the binding of purified HSF1 on HSE. HSF1 (2 μM) was preincubated with AZD (5 μM) and was titrated with increasing concentration HSE (0-10μM) at room temperature. This showed that the quenching of HSF1 fluorescence was 15% greater in the presence of AZD than in its absence. These binding data were analyzed by the double reciprocal plot as described in Experimental Procedures, and the analysis of the data yielded a linear plot with a dissociation constant of 0.35 μM. These results, therefore, show that AZD indeed facilitates binding of HSF1 to HSE (Figures 5B-5C, S3C-D).

We then examined the alteration in HSF1 fluorescence in the presence of AZD alone to test if AZD can bind by itself with HSF1. To our surprise, addition of AZD enhanced fluorescence intensity of HSF1 as the concentration of AZD was increased (0-10 μM) (Figure 5D). No significant amount of fluorescence was observed when only AZD (0-10 μM) was excited at 280 nm and very small background fluorescence of AZD in buffer was subtracted from that of respective HSF1-AZD complex. Analysis of the emission maxima of HSF1 with the dose of AZD revealed a blue shift i.e., shift of spectra towards the lower wave length (Figures 5D, S4A). Blue shift in emission spectra is a signature of trypophan residues responsible for fluorescence, being embedded in the local environment in the protein under the reaction condition. The observed blue shift can be indicative of formation of an oligomeric structure by HSF1 molecules as well [38]. Taken together these results suggested a conformational change in the HSF1 protein in the presence of AZD. The change was HSF1 specific since AZD, unlike geldanamycin at the same concentration range, did not alter fluorescence of HSP90 (Figures 5E-5F). Geldanamycin, however, changed the fluorescence intensity of HSP90 in the same concentration range in dose dependent manner as expected because of its interaction with the substrate binding pocket of HSP90 chaperone [39]. AZD also did not alter fluorescence intensity of another unrelated protein lysozyme in the same concentration range (Figure S5). Next, the stoichiometry and the dissociation constant (Kd) of the HSF1-AZD interaction were estimated by measuring HSF1 fluorescence enhancement. The titration curve of a fixed amount of HSF1 (2 μM) and various concentrations of ligand (0-10 μM) was analyzed by Scatchard plot as described in Experimental Procedures. This analysis yielded a linear plot with a dissociation constant of 1.35 μM and a stoichiometry of 1 (Figure 5D, inset).

HSF1 has been reported to form homotrimer as an intermediate in its activation pathway [36]. Fluorescence intensity of HSF1 increases almost linearly in dose dependent manner as the AZD concentration increased in the reaction (Figure 5D). We tested whether AZD interacts with homotrimeric or monomeric HSF1 with its disulphide bonds reduced. Interestingly, HSF1 pretreated with heat shock (42°C/5 min) that induces homotrimer formation was insensitive to incubation with increasing concentration of AZD (0-10 μM) suggesting that AZD does not interact with trimerized HSF1 (Figure S6B). DTT was reported to disrupt the disulfide bonds and was shown to neutralize HSF1 activation by heat shock [40]. Incubation with increasing concentration of AZD (0-10 μM) did not reveal any change in the fluorescence intensity of HSF1 (2 μM) when it is pretreated with DTT (Figures S6A). Therefore, similar to heat shock, DTT also prevents interaction of AZD with HSF1. Concentration-dependent effect of AZD (as indicated) and heat shock induced multimerization of HSF1in a cell-free system was revealed by monitoring the kinetics of polymerization by using light scattering at 350 nm. As expected, the presence of DTT in the reaction abolished the polymerization induced by AZD or heat shock (Figure 5G). Formation of oligomeric structure was also tested by analysis of HSF1 interaction with AZD by dynamic light scattering (DLS) studies. As shown an addition of AZD shifted majority of population corresponding to higher molecular weight species indicating formation of oligomeric structure of HSF1 by the compound (Figure S7).

Structural change in HSF1 with the addition of the compound was also tested by recording the far-UV CD spectra. A plot of negative ellipticity versus wavelength showed a net decrease in α-helical content in the structure by more than 6 % compared to 4% by heat shock. Random coil content in the protein was found to be increased by more than 5% following AZD treatment compared to 2% by heat shock (Figure S4B and Table S1).

## DISCUSSION

Medicinal plants have been used widely to treat various diseases in traditional medicine systems, although the underlying principles of their actions largely remain unknown. Here we reported our isolation of AZD from *Azadirachta indica* (Neem) as an activator of cellular HSR as measured by upregulation of HSF1 activity. Notably, treatment with AZD led to a significant reduction of protein aggregation and toxicity or symptoms in a mammalian cell and fly models of neurodegenerative diseases. More significantly, AZD showed little toxicity to cells of the organism in the effective concentration range. Neem has been in use in traditional medicine for more than 2000 years for its diverse medicinal properties including its role in neuroprotection and amelioration of Alzheimer's disease [21, 41]. Our present analyses establish the neuroprotective activity of AZD extracted from this plant's seeds. Recently, AZD was shown to inhibit pancreatic α-amylase *in vitro* as well as in a cell based assay exhibiting its potential in anti-diabetic therapy [42].

We showed that the amelioration of neurodegeneration in a fly model of polyQ disorders [33, 43, 54] following AZD treatment (Figure 3) is largely mediated through HSF activation resulting in enhanced expression of HSP70 at transcript as well as protein levels. The results obtained support the idea that AZD exerts its effect through induction of heat shock response. Because antioxidant activity has been reported to reduce protein aggregation induced disorder in fly and mouse models, experiments were carried out to explore if AZD has any antioxidant property or free radical scavenging activity [44, 45]. DPPH and DCFH-DA assay performed showed little free radical scavenging by AZD (Figures S9, panels A-B). Furthermore, AZD has little anti-inflammatory activity as revealed in studies earlier [20, 46].

Our results support the idea that AZD activates DNA-binding competence of HSF1 through stabilizing its homodimeric or higher oligomeric state and that this interaction with HSF1 is specific since AZD, a triterpenoid, did not interact with HSP90ß, or lysozyme (Figures 5A-5F, S5) in the same concentration range and under identical conditions. Further, this interaction with HSF1 is AZD specific since other triterpenoids like celastrol or gedunin, which were previously shown to induce HSP70 transcription, did not physically interact with HSF1 [47]. Our finding of a blue shift in the fluorescence emission spectra and CD analysis (Figure S4A-B, Table S1) also suggest that interaction with AZD induces ordered structural changes in HSF1. While these observations suggest that interaction of HSF1 with AZD is highly specific, they do not seem to involve the cellular activities of proteasome or HSP90 in the HSF1 induction process.

Thus, while AZD protects cellular protein quality control mechanisms by enhancing levels of molecular chaperones like HSP70, it ensures protein chaperone functions since the proteasome and HSP90 functions remain undisturbed. HSP70 induction under these conditions is expected to efficiently drive clearance of protein aggregates in cells through CHIP-mediated ubiquitination and degradation by the proteasome [48].

Our results suggest that one molecule of AZD binds with one molecule of purified full length HSF1. However, this interaction mode apparently changes when interaction of the compound was tested with only the DNA binding domain (DBD) of HSF1. Scatchard plot analysis (Figure S8A) revealed that one molecule of AZD binds with three molecules of DBD of HSF1 (HSF1_DBD_ homotrimer complex). This result is consistent with molecular modeling of DBD of HSF1, a winged helix protein, with its recognition sequence HSE where three molecules of the protein assembled on the HSE [49]. In our model, consistent with our biophysical data, one molecule of AZD interacts with the asparagine residues on two adjacent HSF1 molecules and the phosphate backbones on the DNA (Figure S8C). Alteration of net solvation energy, as well as electrostatic charge distribution in the complex with the addition of AZD, supports its role in the stability of the HSE-HSF1 complex (Table S2) [50].

Finally, AZD identified by this study is unique in its interaction with HSF1 with high specificity resulting in enhanced binding of HSF1 with HSE. Since it does not interfere with HSP90 or proteasome activity, AZD is expected, and indeed found, to show less general toxicity. Our results support a model in which AZD sequesters the free pool of HSF1 that is normally in dynamic equilibrium with different repressive complexes and converts it to an active conformation (Figure 5H). The long term consequences of interruption of this dynamic equilibrium remain unknown. Future study should address this and the global effects on a cell exposed to AZD. Overall, our results show a great potential of AZD as a lead molecule that can be developed as a long desired small molecule therapeutic for NDs, which take a heavy toll on our society. AZD is also should be valuable for a better understanding of the mechanism of HSF1 function.

## MATERIALS AND METHODS

### Extraction purification and identification of Azadiradione

Air dried 1 kg neem seeds were extracted with methanol (1.5 L) by maceration method for one week. The extracts collected by filtering through Whatman filter paper was dried using rotary vacuum evaporator and stored in the refrigerator until further processing. Fractionation was guided by enhancement of specific activity in a collected fraction by silica gel column chromatography (mesh size 230-400, bed volume ~130 ml, L = 25 cm, D = 4 cm) where mobile phase was chosen by checking movement of the compounds on a thin layer chromatography (TLC) plate. For 50 g of crude extract, 400 ml each of the following solvents mixture was applied: hexane: ethyl acetate (H:EtOAc) 1:0, 8:1, 4:1, 3:1, 2:1, 1:1, and 0:1. Collected fractions were dried in a vacuum evaporator and tested for the 6xHSE GFP Rluc reporter activity. Fractions corresponding to H:EtOAc = 4:1 solvent carried the most activity where TLC showed three spots. The active fraction (20 g) was then applied to fresh silica column of same dimension. Analyses of eluents (400 ml each of H:EtOAc in the ratio 6:1, 5:1, 4:1, 3:1, 2:1, 1:1, and 0:1) located activity in fraction corresponding to H:EtOAc::2:1 (180 mg) with a single major spot determined by TLC and HPLC [C^18^ column, (Dimension 4, 6 X 250 mm) ran with acetonitrile: water (60:40) as the mobile phase with flow rate of 1 ml/min (sample injection volume of 20 μl)]. Analysis of ^1^H, ^13^C, NMR and HRMS (ESI mass) spectral data identified the compound as azadiradione.

### Cell culture and cell viability assay

HCT116, HEK293, and Neuro-2a cells were obtained from ATCC. Cells were grown in DMEM supplemented with 10% FBS (Invitrogen), 1% L-Glutamine, 0.1 mM nonessential amino acid, and 100 U/ml penicillin/streptomycin and maintained at 37°C and under 5% CO_2_.

Cells grown to 70% confluence were added in triplicate with different concentrations of AZD or celastrol or DMSO (vehicle) as required. After desired period of treatment cell viability was determined by MTT method (BD bioscience, India). Net survival values (after subtracting the values obtained with DMSO) were calculated for plotting in a graph.

### DPPH radical scavenging activity

DPPH radical scavenger assay was done by mixing in triplicate vehicle, AZD or ascorbic acid (a reference compound) at different concentrations (1 μM to 10 μM) added with water or ethanol to a fixed volume. The solutions were then incubated with 50 μl of DPPH solution to 1 mM in a total volume of 2 ml for 5 min at room temperature in the dark followed by measuring absorbance at 517 nm.

### Measurement of intracellular ROS

HCT116 cells grown in 12 well plates to ~75% confluence were treated in triplicate with 10 μM arsenic, various concentrations of AZD or vehicle for 12 h. After PBS wash cells were incubated with 20 μM DCFH-DA 30 mins at 37°C in the dark. Cells were washed, and analyzed by flow cytometer. The fluorescence intensity was calculated using the FACSuite software.

### Reverse transcription PCR

Reverse transcription (RT) reaction were performed from 1 μg of total RNA isolated by Trizol reagent (Invitrogen) using the I script Bio-Rad cDNA synthesis kit. Semi-quantitative PCR were performed using various gene-specific primers listed in supplementary material (Table S3). PCR products were resolved in 2% agarose gels and were visualized by ethidium bromide staining.

### Expression and purification of human HSF1protein

The pET15b based construct encodes a codon optimized HSF1 protein epitope tagged with his_6_-at the N-terminus. Expression of the protein was induced in *Escherichia coli* strain BL21 (λ-DE3) transformed with this construct by 1 mM IPTG induction at 15°C for 16 h as described [51]. All subsequent steps were conducted on ice or at 4°C. Briefly, after induction, PBS washed cell pellet was lyzed in lysis buffer (50 mM HEPES pH 7.5, 300 mM sodium chloride, 20 mM imidazole, 0.5 mM PMSF, 1 μg/ml of leupeptine, aprotinine, and pepstatin) by sonication. Clear lysate collected by centrifugation (at 20000xg for 30 min) was bound on the nickel-nitrilotriacetic acid (NTA) agarose beads (Qiagen) prewashed with lysis buffer by incubation for 4 h. As a standard approach 1 ml bead was used for cell lysate obtained from 1 liter culture. After Unbound components were washed off the beads with wash buffer (lysis buffer plus 40 mM imidazole), bead bound his_6_-HSF1 protein was eluted in elution buffer (lysis buffer plus 250 mM imidazole) in 0.25 ml fractions until all proteins were collected. Fractions with relatively large amount of the HSF1 proteins tested by SDS-PAGE analysis were pooled together, dialyzed against the lysis buffer, quick freezed in liquid nitrogen in small aliquots, and stored in −80°C for future use.

### Western blot

Whole cell extracts (WCE) were prepared in RIPA lysis buffer(25 mM Tris-HCL pH 7.4, 150 mM NaCl, 1 mM EDTA, 1 mM EGTA, 1% NP-40, 1% sodium deoxycholate, 2.5 mM sodium pyrophoshphate 1 μg/ml leupeptin, 1 μg/ml aprotonin, 1 μg/ml pepstatin A). WCE (20 μg) was resolved in 10% SDS-PAGE gels and transferred to PVDF membrane. The membrane after blocking with 5% BSA for 30 min at room temperature was probed with a primary antibody as appropriate at dilution of 1:5000 (HSP70; Enzo life science, C92F3A), 1:1000 (Raf1; Cell signaling, 9422), 1:1000 (Akt; Cell signaling C67E7), 1:5000 (HSP90α; Biobharti Life Science), 1:10000 (Ubiquitin, Cell signaling; 3933), 1:1000 (FLAG-M2; Sigma aldrich, F3040), or 1:50,000 (β-actin; Abcam, ab8227) for overnight at 4°C. The membrane was then incubated with the secondary antibody as appropriate at dilution of 1:5,000 (Goat anti mouse IgG-HRP, Santa Cruiz, sc-2005), or 1:5,000 (Goat anti rabbit IgG-HRP, Santa Cruiz, sc-2030) for 1 h at room temperature. After several washes the signals were developed by enhanced chemiluminiscence (ECL, Biorad) method.

### Electrophoretic mobility shift assay

EMSA was done using ^32^p-γ-ATP labeled probe (1 ng/reaction) containing the proximal heat shock element (HSE) taken from the human hsp70A1A promoter. Binding reaction were performed essentially under condition as described earlier except that 50 μg of WCE of HEK293 cells stably expressing FLAG-tagged HSF1 or purified recombinant his_6_-tagged protein (0.5 μM) as described [52]. The recombinant protein was purified by using the method described earlier [51]. The reactions were resolved on a 4% polyacrylamide gels (acrylamide and bis-acrylamide 29:1), dried and autoradiographed.

### Fruit fly stocks

#### Oregon R^+^

Wild-type strain of *Drosophila melanogaster*

#### w^1118^; GMR-GAL4

This is a homozygous viable transgenic line in which the yeast *Gal4* gene is downstream of a multimerized copy of the binding site of the *Drosophila* Glass transcription factor (inserted is present on chromosome 2). Glass multimer reporter, is predominantly expressed in all differentiating cells posterior to the morphogenetic furrow in the developing eye discs [53].

#### w^1118^; UAS-127Q [43]

In this stock a transgene carrying 127 tandem repeats of trinucleotide CAG flanked by HA tag placed downstream of the *UAS* promoter on chromosome 2. Homozygous of this transgenic fly is viable without any apparent phenotype. The stock was obtained from Dr. Parsa Kazemi-Esfarjani (University at Buffalo, New York). External morphology of adult eyes following the desired treatment and desired genotype was examined in etherized flies under a Zeiss Stemi SV6 stereo binocular microscope and the eye images were recorded using a Sony Digital Camera (DSC-75).

### Nail polish imprints

A transparent nail polish used to create a replica of the external surface of the eye and subsequent examination by DIC microscopy as described [54]. The eye imprints were examined microscopically using 20X DIC optics in Nikon E800, with an attached Nikon DXM 1200 digital camera.

### Phototaxis assay

This assay was performed with a Y maze comprising of a Y shaped glass tube of 12 mm diameter with each arm of Y being 30 cm long. One arm of the Y maze was covered with black paper to make dark while the arm was left uncovered to serve as lighted chamber. The 1 day, 5 days and 10 days old flies of the desired genotype were subjected to the phototaxis assay for 1 min and the total numbers of flies in each arm were counted [55]. At least three trials were performed for each assay, and the experiment was done in multiple replicates of 75 flies per assay.

### Microscopy and image analysis

For recoding the external morphology of adult eyes, flies of the desired genotype were etherized, and their eyes photographed using a Sony Digital Camera (DSC-75) attached to a Zeiss Stemi SV6 stereo binocular microscope for examining the external morphology. For light microscopy, a Nikon E800 microscope was used with appropriate filter combinations. The images obtained were recorded with a Nikon DXM 1200 digital camera. The different objectives used were 10X (0.3NA, Plan Fluor), 20X (0.5NA, Plan, Fluor) or 60X oil (1.4NA, Plan, Apo). Eye discs from *GMR-GAL4 > 127Q* late third instar larvae reared on DMSO or AZD supplemented food were co-immunostained with 7Fb and anti-haemagglutinin ab for Hsp70 and polyQ IBs, respectively, and examined by confocal microscopy as described earlier [54].

### HSF1 multi-merization assay

HSF1 (2 μM) was mixed with different concentrations of AZD (4- and 8 μM), with or without 1 mM DTT and incubated under heat shock condition. The rate and the extent of the multimerization reaction were monitored by light scattering at 350 nm using a Jasco V-630 (Japan) spectrophotometer.

### Fluorescence study

Absorbance measurements were performed in a JASCO (Tokyo, Japan) V-530 UV-visible spectrophotometer equipped with a Peltier temperature control system, using a cuvette of 1cm path length. All fluorescence measurements were performed using a fluorescence spectrophotometer (PhotoTechnology Inc. USA, Model QM-4CW) equipped with a Peltier temperature control system. Fluorescence data were corrected for the inner filter effect

F = F_obs X_ antilog [(A_ex_ +A_em_) /2]

Where A_ex_ is the absorbance at the excitation wavelength andA_em_ is the absorbance at the emission wavelength

### AZD and HSF1 interaction study

The binding of the ligand to the protein was monitored by enhancement or quencing of protein intrinsic fluorescence in the presence of AZD. Protein (2 μM) (HSF1/Lysozyme/HSP90) was titrated with ligand (0-10 μM) of at room temperature. The excitation wavelength was 280 nm and emission wavelength range was set to 310 nm to 400 nm. The maximum intensity was recorded from each wavelength spectrum for calculation of binding parameters. The dissociation constant K_d_ was calculated from the Scatchard plot according to

r / L_free_ = (r /K_d_)-(n/K_d_)

Where, r is the ratio of the concentration of bound ligand to the total protein concentration and n: represents the maximumnumber of binding sites [56]

The binding of the ligand to the protein was also studied under different condition. We first pre-incubated HSF1 (2 μM) at 42°C for 30 minutes to allow trimerization and then measure the fluorescence of that trimerize HSF1 by titration with ligand (0-10 μM) at room temperature. In another condition, we pretreated HSF1(2 μM) with 1 mM DTT at room temperature and then measure the fluorescence of protein by titrated with ligand (0-10 μM) at room temperature.

### Effect of AZD on HSF1-DNA interaction

The binding of the DNA to the protein was monitored by quenching of protein fluorescence in the presence of DNA. HSF1 (2 μM) was titrated with increasing concentration (0-10 μM) of DNA at room temperature and intrinsic fluoresecence spectrum of mixture was recorded at each concentration of DNA. To understand the effect of AZD on HSF1-DNA interaction, we first pre-incubated 5 μM AZD and 2 μM HSF1for 5 min at room temperature and after that increasing concentration of DNA (0-10 μM) was titrated with that HSF1-AZD complex at room temperature. The apparent decrease in the fluorescence values in the presence of varying concentrations of DNA were corrected for the inner filter effect. The fraction of binding sites (X) occupied by DNA was determined using an equation X = (F0-F)/Fmax [56], where F0 is the fluorescence intensity of HSF1 in the absence of DNA, F is the corrected fluorescence intensity of tubulin in the presence of DNA, and Fmax is calculated from the plot of 1/(F0-F) versus1/[DNA] graph and extrapolating 1/[DNA] to zero. The dissociation constant (Kd) was determined using the relationship, (1/X) = 1 + K_d_/L_f_, where L_f_ represents free DNA concentration, and L_f_ = C -X[Y], where C is total concentration of DNA and [Y] is the molar concentration of DNA-binding sites, assuming a single binding site per HSF1.

### Molecular modeling and docking

The DNA binding domain of HSF1 is docked with another two similar domains to form protein-protein docked trimeric structures. The trimeric HSF1 form was then docked with the double helical DNA with nucleotide sequence 5′-GGCGAAACCCCTGGAATATTCCCGACCTGGCAGC-3′-using Z-Dock server. DNA protein complex then was docked with Azadiradione in Glide module using standard precision (SP) mode (Glide, version 5.5, Schrodinger, Inc., New York, NY, 2009). The grid was prepared to cover the entire structure of DNA with dimensions of 80×80×80 Å.

### Adaptive poisson-boltzmann solver calculations

The electrostatic charge distributions of the protein were calculated using Electrostatic potential surface tools in Schrodinger. Atomic charge distribution, dielectric properties and electrostatic properties of protein can be well correlated using this model. The solute and solvent dielectric constant used for Poisson-Boltzmann settings were 1 and 80 respectively.

## SUPPLEMENTRAY MATERIAL


