# Supplementary material for: Azadiradione ameliorates polyglutamine expansion disease in Drosophila by potentiating DNA binding activity of heat shock factor 1
Source: Oncotarget. 2016 Oct 26;7(48):78281–96. doi: 10.18632/oncotarget.12930 (PMC5346638; doi:10.18632/oncotarget.12930)
Supplement: Supplementary file 1 [file oncotarget-07-78281-s001.pdf]

Azadiradione ameliorates polyglutamine expansion disease in *Drosophila* by potentiating DNA binding activity of heat shock factor 1

Supplementray Material

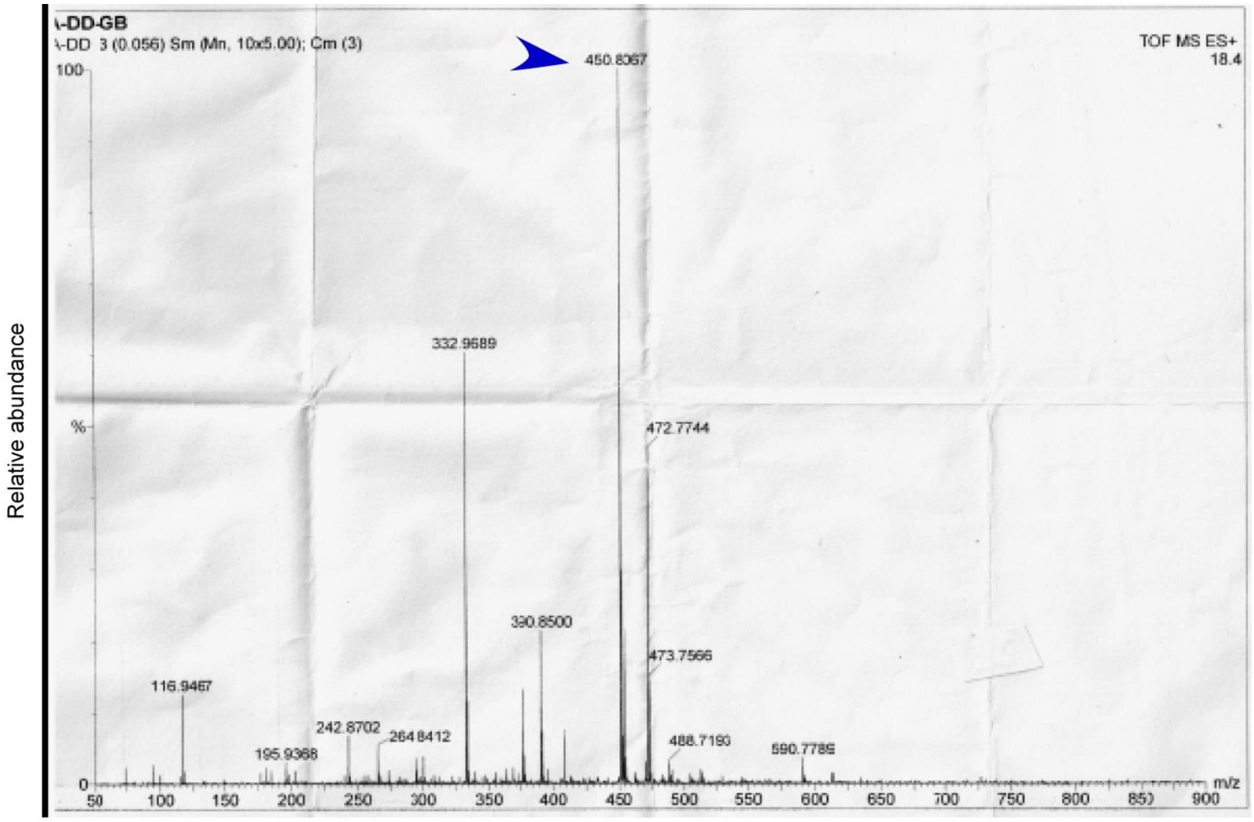

Figure S1A

Figure S1A: ESI-MS spectra of HPLC purified peak fraction. Expected mass of the main peak is indicated (450.8067).

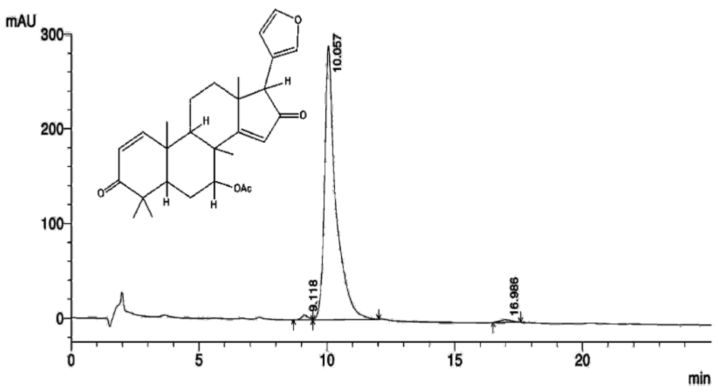

Figure S1B

Figure S1B: Elution profile of HSF1 sensitive activity coincides with that of azadiradione (AZD) from C18 column (dimension 4.6 mmx250 mm). Mobile phase: acetonitrile with 40% water. Sample injection volume: 20 µl.

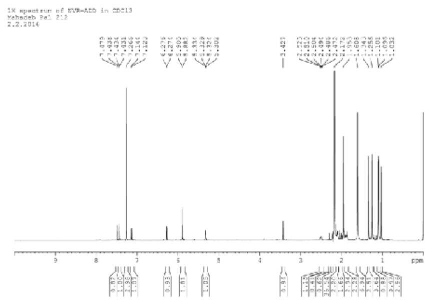

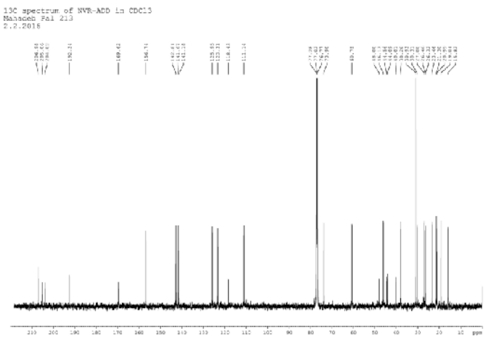

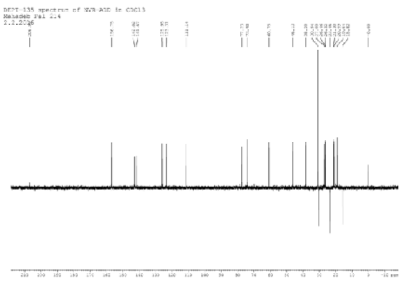

Azadiradione

ESI-MS (m/z): 472.8 ([M+Na]$^+$), 450.8 ([M+H]$^+$).

^{1}H nmr (500 MHz, CDCl$_3$); δ(ppm): 7.47 (1H, m, H-21), 7.43 (1 H, m, H-23), 7.14 (1 H, d, *J* = 10.5 Hz, H-1), 6.27 (1 H, d, *J* = 10.0 Hz, H-2), 5.90 (1 H, s, H-22), 5.88 (1 H, s, H-15), 5.33-5.30 (1 H, m, H-7), 3.42 (1 H, s, H-17), 2.52-2.47 (1H, m, H-9), 2.21 (1 H, m, H-5), 1.95 (3 H, s,-COCH$_3$), 1.34 (3 H, s, H-30), 1.25 (3 H, s, H-19), 1.10 (3 H, s, H-29), 1.09 (3 H, s, H-28), 1.03 (3 H, s, H-18).

^{13}C nmr (125 MHz, CDCl$_3$); δ(ppm): 205.06 (C-16), 204.02 (C-3), 192.34 (C-14), 169.62 (C-COCH3), 156.74 (C-1), 142.81 (C-23), 141.67 (C-21), 125.95 (C-2), 123.31 (C-15), 118.43 (C-20), 111.14 (C-22), 73.90 (C-7), 60.75 (C-17), 48.00 (C-13), 46.13 (C-5), 44.56 (C-8), 44.09 (C-4), 40.01 (C-10), 38.20 (C-9), 30.31 (C-12), 27.00 (C-28), 26.46 (C-18), 26.32 (C-30), 23.46 (C-6), 21.30 (C-29), 20.99 (C-COCH$_3$), 19.04 (C-19), 15.82 (C-11).

Figure S1C: The NMR (^{1}H, ^{13}C and DEPT-135) spectra along with the NMR values.

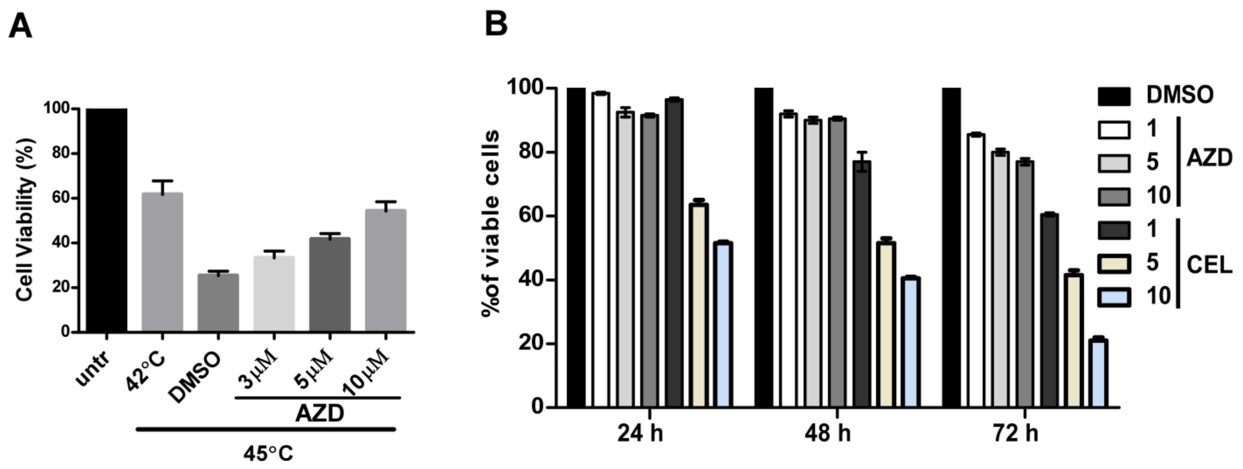

Figure S2

Figure S2: Pretreatment with AZD protects cells from subsequent challenge with lethal heat shock and toxicity of Neuro-2a cells when incubated for 3 days. A) HEK293 cells were either left untreated or treated with DMSO, or indicated concentrations of AZD for 4 h, or 42°C for 1 h followed by subjected to exposure to heat shock at 45°C for 1 h. Cells were then replenished with fresh growth medium and allowed to grow in a standard growth condition. The next morning the viability of cells were measured by MTT assay and plotted as a bar graph. **B.** Bar graph comparing the viability of Neuro-2a cells to a range of concentrations of AZD versus celastrol estimated by MTT assay.

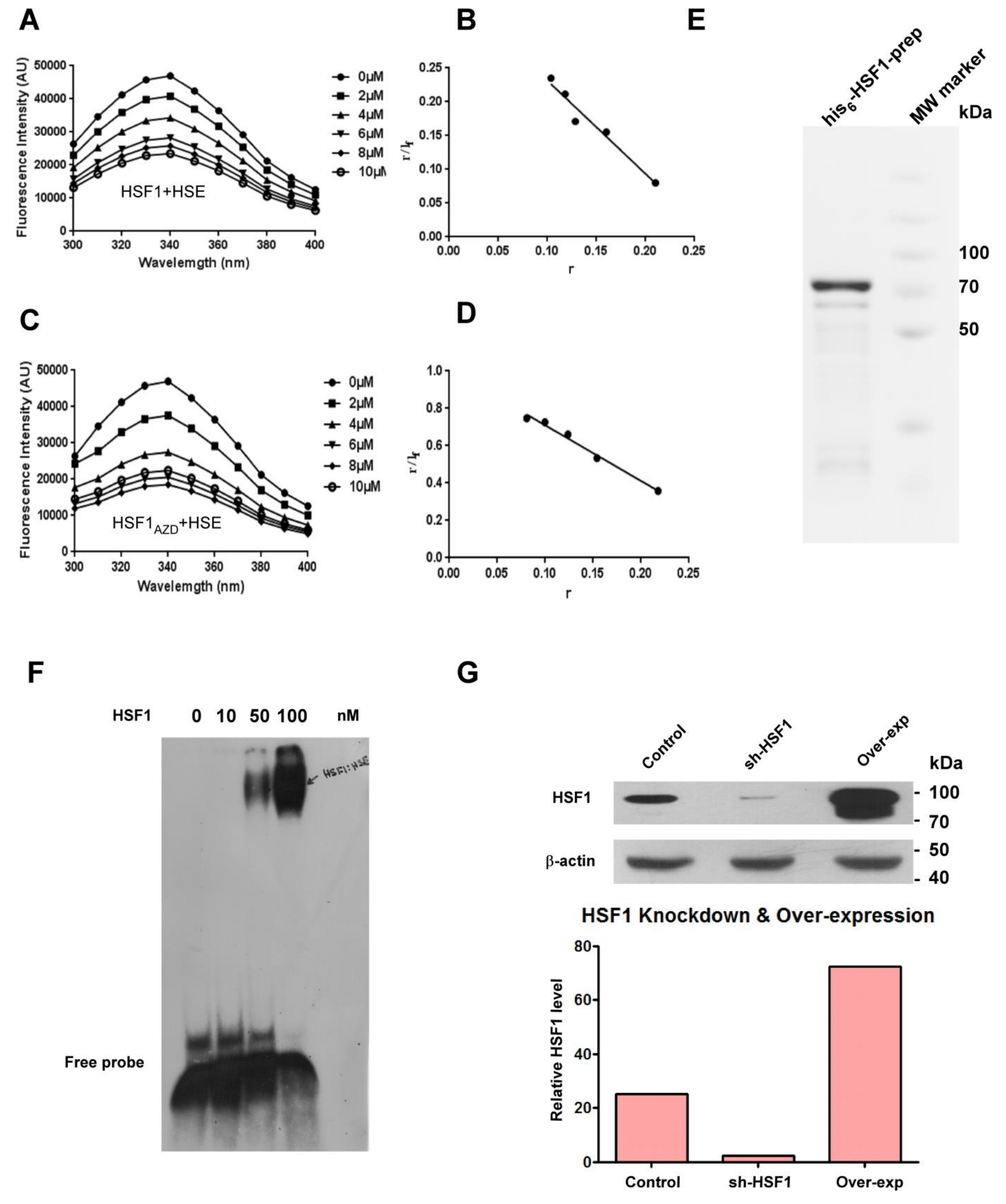

Figure S3

Figure S3:

Fluorometric study of HSF1 interaction with recognition sequence HSE with and without AZD: **A.** Plot of fluorescence emission spectra of HSF1 (2 μM) with indicated concentrations of HSE

(0-10 µM). **B.** Scatchard plot to estimate the stoichiometry of HSF1 and HSE binding. **C.** The plot of fluorescence emission spectra of HSF1 with indicated concentrations of HSE (0-10 µM) supplemented with 5µM AZD, **D.** Scatchard plot to estimate the stoichiometry of HSF1 and HSE binding in the presence of AZD. **E.** An aliquot (~0.5 µg) of his$_6$-HSF1 (HSF1-prep) purified by nickel-NTA beads resolved by SDS-PAGE visualized by coomassie brilliant blue staining. **F.** Autoradiogram representing an electrophoretic mobility shift assay (EMSA) with radiaolabelled HSE (1 ng/reaction) using non-codon optimized HSF1 protein (encoded by native cDNA) purified from bacterial overexpressing strain. Concentrations (nM) of HSF1 added per reaction is shown on the top. HSE:HSF1 complex indicated. **G.** Immunoblot showing levels of expression of HSF1 in whole cell extracts of HEK293 cells normal (control), treated with shRNA against HSF1 (shHSF1) or stably overexpressing FLAG-HSF1 (over-exp) under the control of CMV promoter using anti-HSF1 antibody. β-actin levels were determined as loading control. Lower panel: Bar graph showing the intensity of bands determined by densitometric scanning.

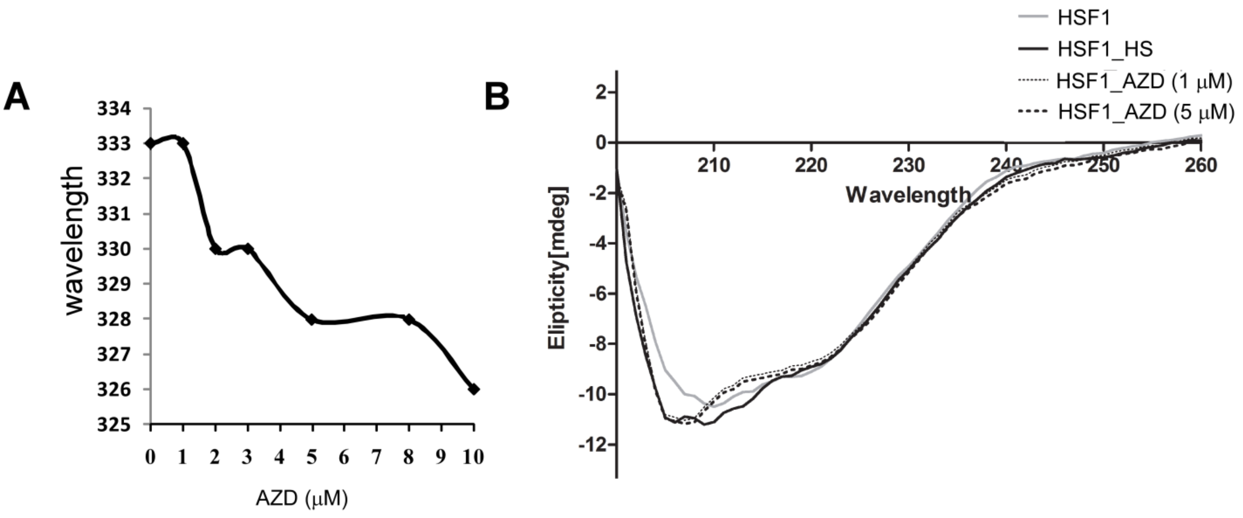

Figure S4

Figure S4: AZD interaction with HSF1 induces a structural change in the HSF1 protein determined by fluorometric and CD studies. **A.** The addition of AZD to HSF1 (2 µM) protein induces a blue shift in the emission spectra shown by plotting the change of emission maxima with increasing concentration of AZD. **B.** CD analysis to estimate the alteration of secondary

structure in the HSF1 (2 μM) protein upon incubation with indicated concentration of AZD (also see Table S1). The ellipticity changes are plotted against the wavelength. HSF1 incubated with the vehicle or treated with either two concentrations of AZD or HS.

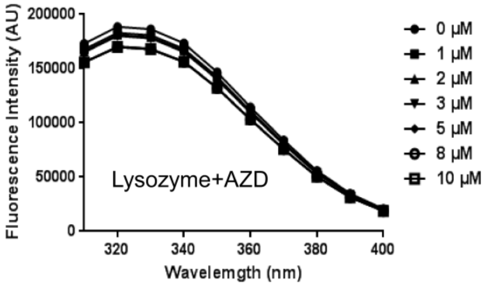

Figure S5

Figure S5: AZD does not interact with lysozyme in vitro. The plot of fluorescence emission spectra of lysozyme (2 μM) with increasing concentrations of AZD indicated.

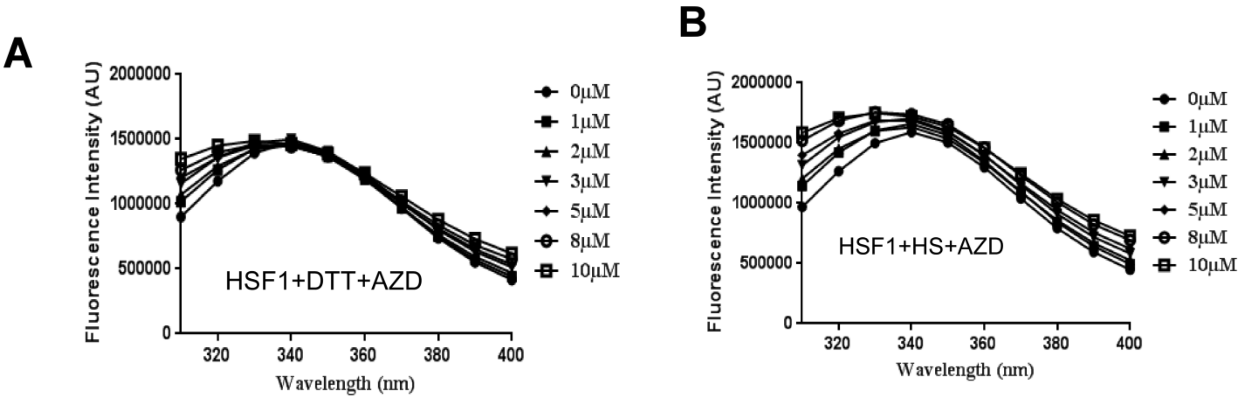

Figure S6

Figure S6: Interaction of AZD with HSF1 like HS is sensitive to DTT.

A. The plot of fluorescence emission spectra of HSF1 (2 μM) incubated with indicated concentrations of AZD (0-10 μM) with DTT (2 mM) treatment. **B.** The plot of

fluorescence emission values of HSF1 (2 µM) incubated with indicated concentrations of AZD (0-10 µM) with prior treatment with HS (42°C/30 min).

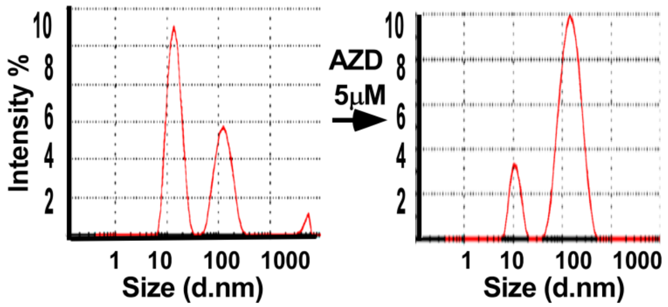

Figure S7

Figure S7: Plot of hydrodynamic radius of purified HSF1 determined by dynamic light scattering (DLS) assay with and without the addition of AZD. HSF1 normally occurred in solution with forms represented by two distinct peaks of hydrodynamic radii 15 nm and 150 nm without AZD. The addition of AZD converted the majority of the HSF1 population (peak) to the higher particle size.

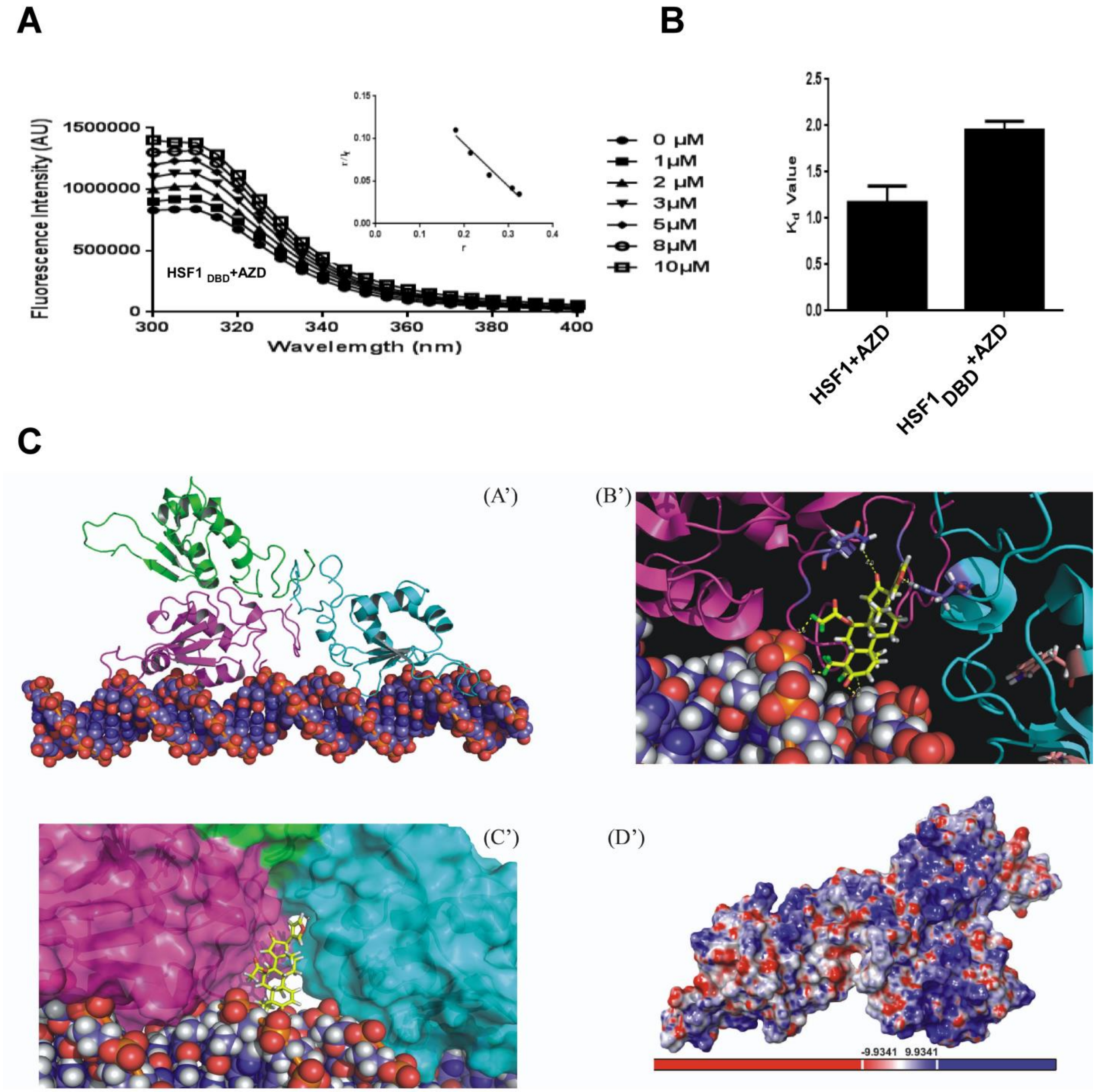

Figure S8

Figure S8

AZD docks in a complex comprising three molecules of HSF1-DNA binding domain of HSF1 bound to HSE. **A.** Incubation with AZD (0-10μM) enhances fluorescence emission of ~35 KD DNA binding domain (DBD) of HSF1 (2 μM, $HSF1_{DBD}$). (inset: Scatchard Plot of AZD binding

to DBD of HSF1). **B.** Bar diagram representing the kd values of AZD binding to HSF1 full length and $HSF1_{DBD}$. **C.** (A') Trimeric $HSF1_{DBD}$ bound to DNA holding a molecule AZD. The DNA and $HSF1_{DBD}$ protein segment are bridged by AZD (B'). One side of the AZD is stabilized by hydrogen bonding with two asparagine residues of the $HSF1_{DBD}$ and the other side is stabilized by the CH_3 (C-H-O) interactions with DNA phosphate backbone. (C') Surface representation of the $HSF1_{DBD}$ docked with DNA, in the cavity of DNA-protein complex AZD is placed by standard precision docking. (D') Coulombic surface area of trimeric $HSF1_{DBD}$. The surface potential is calculated on Schrodinger software with the range of -9.9 KT/e to +9.9 KT/e (also Table S2)

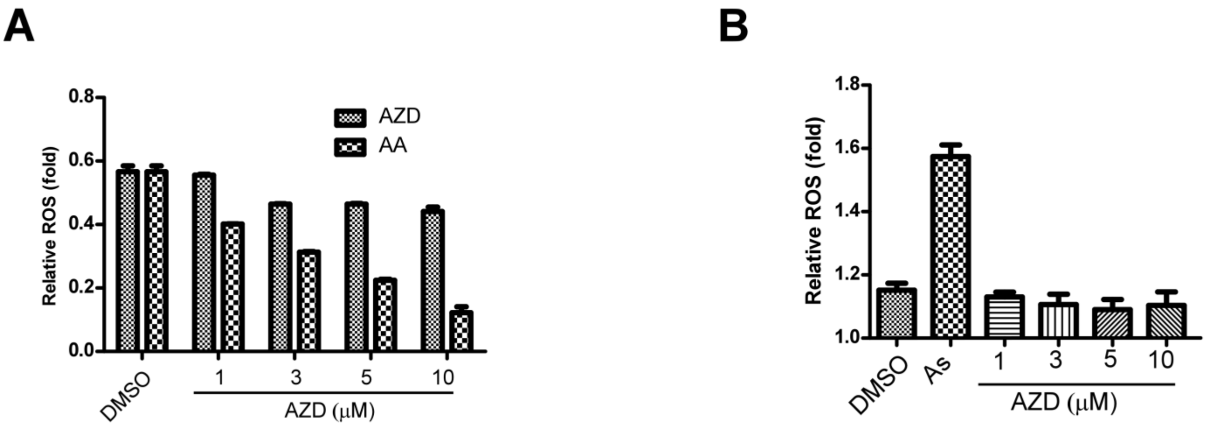

Figure S9

Figure S9: AZD showed weak ROS scavenging activity measured by DPPH or DCFH-DA assays, respectively. **A.** DPPH assay was done with indicated concentration of AZD using ascorbic acid(AA) as a positive control. **B.** DCFH-DA assay was done in HCT116 cells using arsenic (10 µM) as the internal control. DMSO was used as the vehicle control.

Table S1: Alteration of the secondary structure of HSF1 induced by treatment with AZD or heat shock determined by CD spectroscopy. The data presented in the table were obtained by analyzing the results by cdnn software

Table 1

HSF1

	180-260 nm	185-260 nm	190-260 nm	195-260 nm	200-260 nm	205-260 nm	210-260 nm
Helix	n.d.	n.d.	n.d.	n.d.	30.40%	33.10%	31.50%
Antiparallel	n.d.	n.d.	n.d.	n.d.	9.70%	8.00%	8.80%
Parallel	n.d.	n.d.	n.d.	n.d.	9.20%	9.10%	9.20%
Beta-Turn	n.d.	n.d.	n.d.	n.d.	17.70%	16.80%	17.20%
Rndm. Coil	n.d.	n.d.	n.d.	n.d.	32.40%	33.30%	34.40%
Total Sum	-	-	-	-	99.40%	100.30%	101.00%

HSF1_HS

	180-260 nm	185-260 nm	190-260 nm	195-260 nm	200-260 nm	205-260 nm	210-260 nm
Helix	n.d.	n.d.	n.d.	n.d.	29.40%	31.80%	29.60%
Antiparallel	n.d.	n.d.	n.d.	n.d.	10.10%	8.30%	9.30%
Parallel	n.d.	n.d.	n.d.	n.d.	9.40%	9.50%	9.70%
Beta-Turn	n.d.	n.d.	n.d.	n.d.	17.90%	17.00%	17.60%
Rndm. Coil	n.d.	n.d.	n.d.	n.d.	32.70%	34.30%	35.70%
Total Sum	-	-	-	-	99.60%	100.90%	101.90%

HSF1_1µM AZD

	180-260 nm	185-260 nm	190-260 nm	195-260 nm	200-260 nm	205-260 nm	210-260 nm
Helix	n.d.	n.d.	n.d.	n.d.	28.90%	30.50%	28.00%
Antiparallel	n.d.	n.d.	n.d.	n.d.	10.30%	8.60%	9.80%
Parallel	n.d.	n.d.	n.d.	n.d.	9.60%	9.90%	10.10%
Beta-Turn	n.d.	n.d.	n.d.	n.d.	18.00%	17.20%	17.90%
Rndm. Coil	n.d.	n.d.	n.d.	n.d.	33.40%	35.30%	36.90%
Total Sum	-	-	-	-	100.30%	101.50%	102.80%

Table S2: Alteration of energy due to binding of AZD in the $HSF1_{DBD}$-HSE complex calculated by use of Adaptive Poisson-Boltzmann Solver (APBS) procedure. The electrostatic charge distributions of the protein were calculated using electrostatic potential surface tools in Schrodinger. Atomic charge distribution, dielectric properties and electrostatic properties of a protein can be well correlated using this model. The solute and solvent dielectric constant used for Poisson-Boltzmann settings were 1 and 80 respectively. The calculation for coulombic surface area is carried out at 298K.

Energy (kJ/mole)	Monomer	Trimer	Trimer+DNA	DNA	Trimer + Ligand	Trimer + DNA+Ligand
Stretch Energy	813.38	2574.94	4304.97	1740.48	2737.94	4402.70
Bend Energy	917.50	5175.64	9743.52	4157.75	5307.63	9981.08
Torsion Energy	1758.20	5336.34	8492.64	3042.55	5601.88	8745.71
Improper Torsion	19.31	61.42	78.39	8.36	62.66	79.67
Van der Waal Energy	-733.85	1363601.75	7137412	2186.60	1363990.63	7137406
Electrosostatic Energy	-18321.23	-55244.16	4633.00	77660.76	-55465.61	4445.19
Solvation Energy	-7002.51	-19785.05	-129705.60	-128737	-19845.10	-129613.73
Total Energy	-22549.19	1301721	7034959	-39940.3	1302390	7035446.50

Table S3: Nucleotide sequence of DNA oligonucleotides used as primers in RT-PCR.

Human HSP70 (forward)	TGCCGGTTCCCTGCTCTCTG
Human HSP70 (reverse)	TGCCGGTTCCCTGCTCTCTG
Human HSP27 (forward)	TAGAGACCTCAAACACCGC
Human HSP27 (reverse)	GTCATGCTGGCTGACTCT
Human HSF1(forward)	GCCTTCCTGACCAAGCTGT
Human HSF1(reverse)	GCCATGTTGTTGTGCTTGAA
Human GAPDH (forward)	CGACCACTTTGTCAAGCTCA
Human GAPDH (reverse)	TTCCTCTTGTGCTCTTGCTG
Drosophila HSP70 (forward)	AGCCGTGCCAGGTTTG
Drosophila HSP70 (reverse)	CGTTCGCCCTCATACA
Drosophila RP49 (forward)	AGCGCACCAAGCACTTCATCCGCCA
Drosophila RP49 (reverse)	GCGCACGTTGTGCACCAGGAACTTC
Mouse HSP70 (forward)	AGTGACCTTCGACATCG
Mouse HSP70 (reverse)	CTCCTGCAGCATGCGCTCGAT
Mouse GAPDH (forward)	AAGGTCATCCAGAGCTGAA
Mouse GAPDH (reverse)	CTGCTTCACCACCTTCTTGA
